# *Mycobacterium colombiense* and Pseudotuberculous Lymphadenopathy

**DOI:** 10.3201/eid1504.081436

**Published:** 2009-04

**Authors:** Katariina Vuorenmaa, Iskandar Ben Salah, Vincent Barlogis, Hervé Chambost, Michel Drancourt

**Affiliations:** Université de la Méditerranée, Marseille, France (K. Vuorenmaa, I. Ben Salah, M. Drancourt); Assistance Publique des Hôpitaux de Marseille–Fédération de Microbiologie Clinique Hôpital la Timone, Marseille (K. Vuorenmaa, I. Ben Salah, M. Drancourt); Hôpital d’Enfants la Timone, Marseille (V. Barlogis, H. Chambost)

**Keywords:** Mycobacterium colombiense, lymphadenopathy, caseous necrosis, letter

**To the Editor:** *Mycobacterium colombiense* is a new species belonging to the *M. avium* complex (MAC). It is characterized by a unique internal transcribed spacer sequence and causing respiratory tract and disseminated infection in HIV-infected patients in Colombia (*1*). We report clinical and histologic features of lymphadenopathy resulting from *M. colombiense* infection.

A 25-month-old girl with an unremarkable medical history was hospitalized in the pediatric department of Timone Hospital, Marseille, France, due to development of swelling in a right subclavicular lymph node over a 1-month period. A 5-day course of oxacillin, which was administered orally, had been unsuccessful in alleviating the symptoms. The patient’s general condition was excellent, and results of a physical examination were normal, with the exception of a 2-cm hard, immobile, yet painless, noninflammatory, enlarged lymph node. Due to the presence of the enlarged lymph node, a chest radiograph was performed, and results were normal. A hemogram indicated a hemoglobin concentration of 113 g/L, a leukocyte count 8.3 × 10^9^/L consisting of 31% polynuclear neutrophils and 62% lymphocytes, and a normal blood smear. A platelet count indicated a concentration of 389 × 10^9^/L, and the serum lactic dehydrogenase level was 440 UI/L. In addition, no biologic inflammatory syndrome was observed based on the concentration of C-reactive protein (<1 mg/L) and an erythrocyte sedimentation rate of 18 mm/h.

Fine-needle aspiration of the lymph node showed necrosis and mature, activated lymphocytes. These results suggested a possible diagnosis of lymphoma, and a surgical excision biopsy was subsequently performed. Direct microscopic examinations were carried out after results obtained by Gram and Ziehl-Neelsen staining showed that the lymph node was negative for acid-fast bacilli. Histopathologic analysis indicated epithelioid cell granulomas containing giant cells and caseous necrosis without altered leukocytes, all of which are compatible with tuberculosis. Culturing of the biopsy specimen in BACTEC broth (Becton Dickinson, Courtaboeuf, France) at 5% CO_2_ at 37°C yielded acid-fast bacilli after a 7-day incubation period.

After inactivating the cells and extracting the DNA by using a previously described method, we identified the isolate by PCR sequencing of the *rpo*B gene (*2*) and its demonstrated 100% sequence similarity to *M. colombiense* CIP108962^T^ (*1*,*2*). Accordingly, the isolate exhibited positive urease activity, a distinctive characteristic that differentiates *M. colombiense* from other MAC species (*1*,*2*).

Recently, *M. colombiense* was shown to be responsible for an enlarged lymph node in 1 child from Spain who did not show any evidence of HIV infection (*3*). In that patient, histopathologic examination showed granulomatous adenitis with necrosis. We report that *M. colombiense*–infected lymph nodes also yield clinical and histopathologic features evocative of tuberculosis. Indeed, MAC organisms remain the most prevalent agents demonstrated in diseased lymph nodes in children (*4*).

Because modern molecular tools used for the description of emerging MAC species have not been available in most previous reports, the real prevalence of *M. colombiense* may have been underestimated. In children, *M*. *hemophilum* (*5*), *M. avium* subsp. *avium* (*6*), *M. avium* subsp. *hominissuis* (*7*), *M. lentiflavum* (*8*), *M. bohemicum* (*9*), and *M. simiae* (*10*) have been demonstrated to be responsible for enlarged cervical lymph nodes (Appendix Table). Because management and antimicrobial drug treatment of each of these different infections vary in terms of indication, choice of drugs, and duration, the accurate and rapid identification of the causative *Mycobacterium* species is absolutely necessary. This identification should use PCR sequencing of selected universal molecular targets, including the 16S rRNA and *rpo*B genes (*2*), as illustrated herein.

## Supplementary Material

Appendix Table Mycobacterium spp. implicated in lymphadenopathy in children, France*
