# Supplementary material for: Mycobacterium colombiense and Pseudotuberculous Lymphadenopathy
Source: Emerg Infect Dis. 2009 Apr;15(4):619–20. doi: 10.3201/eid1504.081436 (PMC2671429; doi:10.3201/eid1504.081436)
Supplement: Appendix Table — Mycobacterium spp. implicated in lymphadenopathy in children, France* [file 08-1436_appT-s1.pdf]

Appendix Table. *Mycobacterium* spp. implicated in lymphadenopathy in children, France*

	Clinical features			Microbiologic tests			Histopathology				
Mycobacterium spp.	No. patients	Treatment	Outcome	Direct examination†	Culture	Molecular identification	Ring of epithelial cells	Giant cells	Caseous necrosis	Altered leukocytes	Reference
M. tuberculosis				±	+	IS*6110*	+	+	+	–	(*6*)
M. avium	183	NR	NR	NR	+	NR	+	+	NR	NR	(*7*)
M. avium subsp. *hominissuis*	34	NR	NR	NR	+	IS*1245*	NR	NR	NR	NR	(*5*)
M. haemophilum	39	NR	NR	+	+	16S–23S rDNA	+	+	+	–	(*8*)
M. lentiflavum	3	SE	Cured	+	+	16S rRNA	+	+	NR	+	(*9*)
M. bohemicum	4	SE/C, R	Cured	±	+	16S rRNA	+	+	+	–	(*10*)
M. simiae	1	SE	Cured	+	+	HPLC	+	+	+	–	(*3*)
M. colombiense	1	SE	Cured	+	+	16S rRNA	+	+	+	–	This study
	1	SE	Cured	–	+	*rpo*B	+	+	+	–	

*IS, insertion sequence; NR, not reported; SE, surgical excision; C, clarithromycin; R, rifampin; NR, not reported; HPLC, high-performance liquid chromatography.
†Zielhl-Nelsen staining.
